# Mylohyoid hemispasm in a patient with hypoglossal nerve stimulation

**DOI:** 10.1002/ccr3.1650

**Published:** 2018-06-21

**Authors:** Norbert Brüggemann, Armin Steffen

**Affiliations:** ^1^ Department of Neurology University of Lübeck Lübeck Germany; ^2^ Institute of Neurogenetics University of Lübeck Lübeck Germany; ^3^ Department of Otorhinolaryngology University of Lübeck Lübeck Germany

**Keywords:** hypoglossal nerve stimulation, mylohyoid muscle, myoclonus, sleep apnea

## Abstract

Unilateral hypoglossal nerve stimulation may be associated with myoclonus of the mylohyoid muscle.

Unilateral hypoglossal nerve stimulation is an emerging therapy for patients with obstructive sleep apnea who do not sufficiently respond to CPAP therapy. We report a 49‐year‐old patient with CPAP‐resistant sleep apnea who developed myoclonus of the mylohyoid muscle 3 years after the implantation of a hypoglossal nerve stimulator.

Unilateral hypoglossal nerve stimulation prevents the upper airway from collapsing in severe sleep apnea.^1^ This 49‐year‐old patient underwent implantation 3 years ago. He complained about twitches of the ipsilateral neck area that have never occurred before. The twitches were consistent with myoclonus of the mylohyoid muscle and resolved spontaneously over time. Technical failure was unlikely as the rate and amplitude of the myoclonus were independent of the rate and presence of stimulation. Further, the mylohyoid muscle is not innervated by the hypoglossal but the mylohyoid nerve that is a branch of the trigeminal nerve.^2^ The extension cable may have traversed the mylohyoid nerve and may have predisposed to the formation of ephapses that resulted in spontaneous discharges. The spontaneous remission, however, challenges the concept of a direct and persistent contact between nerve and extension cable (Video S1).

## CONFLICT OF INTEREST

None declared.

## AUTHOR CONTRIBUTION

NB: involved in the acquisition of data, supervised the study, analyzed and interpreted the data, wrote the first draft. AS: involved in the acquisition of data, supervised the study, analyzed and interpreted the data, and critically revised the manuscript for important intellectual content.
