# Supplementary material for: Mylohyoid hemispasm in a patient with hypoglossal nerve stimulation
Source: Clin Case Rep. 2018 Jun 21;6(8):1657–8. doi: 10.1002/ccr3.1650 (PMC6099061; doi:10.1002/ccr3.1650)
Supplement: Supplementary file 2 [file CCR3-6-1657-s002.docx]

**Video legend**

In the first segment, the stimulator is switched off. Myoclonus of the anterior neck region is nevertheless present and could be assigned to the mylohyoid muscle. In the second part, the stimulator is switched on and results in bulging of muscles of the anterior neck during inspiration, and independent myoclonus.
